# The Dialectics of Altered Experience: How to Validly Construct a Phenomenologically Based Diagnosis in Psychiatry

**DOI:** 10.3389/fpsyt.2022.867706

**Published:** 2022-04-12

**Authors:** Guilherme Messas, Lívia Fukuda, K. W. M. Fulford

**Affiliations:** ^1^Santa Casa de São Paulo School of Medical Sciences, São Paulo, Brazil; ^2^Collaborating Centre for Values-Based Practice, St Catherine's College, Oxford, United Kingdom; ^3^Philosophy Faculty, St Catherine's College, University of Oxford, Oxford, United Kingdom; ^4^Philosophy and Mental Health, University of Warwick, Coventry, United Kingdom

**Keywords:** psychiatric diagnosis, dialectics, phenomenological psychopathology, dialectical phenomenology, phenomenological psychiatry, hermeneutics, value-based psychiatry, validity

## Abstract

In this paper, we present how a dialectical perspective on phenomenological psychopathology, called Dialectical Phenomenology (DPh), can contribute to current needs of psychiatric diagnosis. We propose a three-stage diagnostic methodology: first- and second-person stages, and synthetic hermeneutics stage. The first two stages are divided into a pre-dialectical and a dialectical phase. The diagnostic process progresses in a trajectory of increasing complexity, in which knowledge obtained at one level is dialectically absorbed and intertwined into the next levels. Throughout the article, we offer some examples of each step. In overall, the method starts off from the patient's own narrative, proceeds to two stages of phenomenological reduction designed to guarantee the scientific validity of the object, and concludes with a hermeneutical narrative synthesis that is dialectically composed of the patient's and psychopathologist's shared narratives. At the end of this process, the initial first-person narrative is transformed into a specific scientific object, a full dialectical phenomenological psychiatric diagnosis. This form of diagnosis constitutes a comprehensive alternative for an integral assessment of the complexities of human psychological alteration, bringing together both the interpretation of the suffering person and the scientific categories of psychiatry.

## Introduction

Diagnosing disorders is crucial for health care promotion worldwide. The implementation of global health priorities depends ultimately on a wider population having access to diagnoses in all fields of health (1). Mental health is one of the most important topics of this universal agenda because of the great burden that mental disorders impose on all societies. The topic of diagnosis in psychiatry, however, is especially marked by the most diverse of controversies, ranging from doubts regarding the nature of mental disorders and the validity of diagnoses (2, 3) to uncertainties about the role of diagnosis in establishing clinical strategies adjusted to a person-centered psychiatry (4). In general, we could say that criticisms to current mainstream psychiatric diagnosis point out certain shortfalls when it comes to apprehending the complex, ambiguous and dynamic nature of the object of study. Evidently, any imprecision in the diagnostic act in psychiatry has immediate consequences for the provision of mental health care to the population. Thus, an investigation into diagnosis in psychiatry is not only pertinent but of crucial importance for the proper provision of mental health in all societies, with effects that impact the neuroscientific, epidemiological, therapeutic and preventive dimensions, and ultimately public policies.

With the aim of meeting the needs of the 21st century, appeals have increasingly been voiced to incorporate a phenomenological dimension to our understanding of psychiatric disorders. As a contribution to this issue, we have recently proposed that a dialectical perspective on phenomenology, called Dialectical Phenomenology (DPh), could provide conceptual tools that are well-suited to this contemporary agenda in psychiatry (5).

In this article, we intend to advance a presentation of the ways in which DPh can contribute to current psychiatry, focusing particularly on the diagnostic process. Through a presentation of the methodological steps by which a scientifically valid diagnosis is built in phenomenology, we hope to emphasize both the particular meanings of phenomenological diagnoses and the relevance they have to general diagnoses in psychiatry. It is important to emphasize that the DPh diagnostic process does not intend to replace other psychiatric methods. The following proposal seeks to enrich the diagnostic process in psychiatry by enabling scientific diagnoses to get closer to the complexities of clinical reality, bringing together both the core altered experience of the disorder and the way the person assigns value to it.

As DPh involves applying and updating some classic concepts of philosophy to psychopathology, which are then used to determine specific notions of mental disorder, we would like to begin by briefly introducing these founding concepts.

### Dialectics

Although often attributed to the early nineteenth century German philosopher, Georg Hegel, dialectics has a long history in philosophy. Hegel himself positioned his work as building on and critiquing the dialogic form of argument adopted by Plato in his Dialogues. Like dialogue, dialectical argument runs back-and-forth. Plato's Dialogues involved a back-and-forth exchange with his opponents aimed at bringing them to discover for themselves the correctness of his views. Hegel's dialectic method involved a back-and-forth process between opposite concepts with the aim of taking philosophy beyond such traditional dualisms as “necessity and freedom”. Central to Hegel's dialectic was his notion of sublation (“*Aufhebung”)* according to which the back-and-forth between opposites of dialectics is aimed at integration without elimination or reduction. The Hegelian dialectic is often summed up in the familiar triad of thesis-antithesis-synthesis (this terminology is nowadays generally attributed to Hegel's contemporary, and co-national, Johann Gottlieb Fichte).

It is the Hegel/Fichte model of dialectics that we believe offers a basis for a repurposed phenomenology. To be clear, it is not with the specifics of either philosopher's work, nor is it with their wider contributions to the German philosophy of their day, with which we will be concerned. Our focus will instead be on the general features of their dialectic, the back-and-forth movement between opposites aimed at integration without elimination or reduction, that, we will argue, provides a basis for refocusing and extending phenomenology to meet the challenges of twenty-first century psychiatry.

### Dialectics and Phenomenological Psychopathology

Phenomenology, broadly construed, explores the way we experience and make sense of the world around us, our “subjective life world” as it is often called (6). It is not alone in this - psychology and psychoanalysis for example have both provided many important insights into people's subjective life worlds. Phenomenological psychopathology however differs from other disciplines in being concerned with the **pre-reflective** basis of subjective experience. This key feature of phenomenological psychopathology is derived from the founder of contemporary phenomenology, the early twentieth century German philosopher, Edmund Husserl. The idea—expressed metaphorically—is that our direct reflections on the world make sense only to the extent that we view them through a set of “lenses” (not Husserl's term). These lenses are *pre*-reflective and hence we are not normally aware of them.

Psychopathology, so this view suggests, is thus illuminated by phenomenology essentially through the insights that it provides into **disturbances** of these pre-reflective “lenses”. *Dialectical* phenomenological psychopathology is in turn marked out by its focus on disturbances in the *balance* between what may be called (by extension of the above metaphor) *sets* of pre-reflective lenses. This idea originates in the middle years of the twentieth century with the work of German psychiatrist and psychologist, Binswanger (7), later emphasized by the German psychiatrist and phenomenologist Blankenburg (8), and further developed by the Chilean psychiatrist and phenomenologist, Zegers (9). Binswanger, Blankenburg, Dörr-Zegers, and others, started from the observation that the pre-reflective basis of our everyday experiences of the world (our subjective “life world”) is constituted by reciprocal relationships between sets of opposites. These opposites are normally kept in a dynamic balance. Psychopathology, so this observation suggests, thus arises when the requisite balance fails, with one or other component of the set of opposites in question becoming over-dominant, bringing forth an anthropological disproportion (10). Dialectical understanding of psychopathology thus involves **exploring** the failures of normal balancing in the pre-reflective life world of a **given individual** with interventions being targeted at *restoring* the required balance. As we will develop below, the entire diagnostic process entails a search for the dynamic of these opposites. In general, we could say that the investigation of opposites are relevant not only for diagnosis, through the notion of anthropological disproportions (which we will explore in this paper), but also for clinical decision-making, through the notions of ambiguity and of dialectics between values and position (5).

Hence, for DPh, making a diagnosis consists in following a structured methodology of validated procedures to enable the scientific identification of specific pre-reflexive alterations of lived experience, which we will explore further in this article. Although there is a growing body of work on phenomenological psychopathology (11), it seems to us that there is still room to further explore the specific cognitive processes by which conclusions are reached using this approach. Furthermore, it is our understanding that the procedures to be followed when taking a dialectical perspective in phenomenological psychopathology have not yet been elucidated. To address these gaps, this article constitutes a first attempt to present the general features of the methodological procedures to be observed when employing DPh to make a psychiatric diagnosis.

## The Stages of Dialectical Phenomenological Diagnosis

In this section, we describe the stages of a DPh diagnostic procedure. Although, for didactic reasons, the stages are presented separately, it is worth noting that they overlap, intertwine, and involve higher-level syntheses (Hegelian sublations), so that each stage inevitably contains something of the previous ones. Strictly speaking, the proposed DPh diagnostic method enables distinct perspectives in the patient-psychopathologist relationship to be taken into account, giving precedence to different perspectives at different stages in the process, although we posit that ultimately the second-person perspective should take precedence for the proper scientific use of the method and an adequate diagnosis.

**Figure 1:**
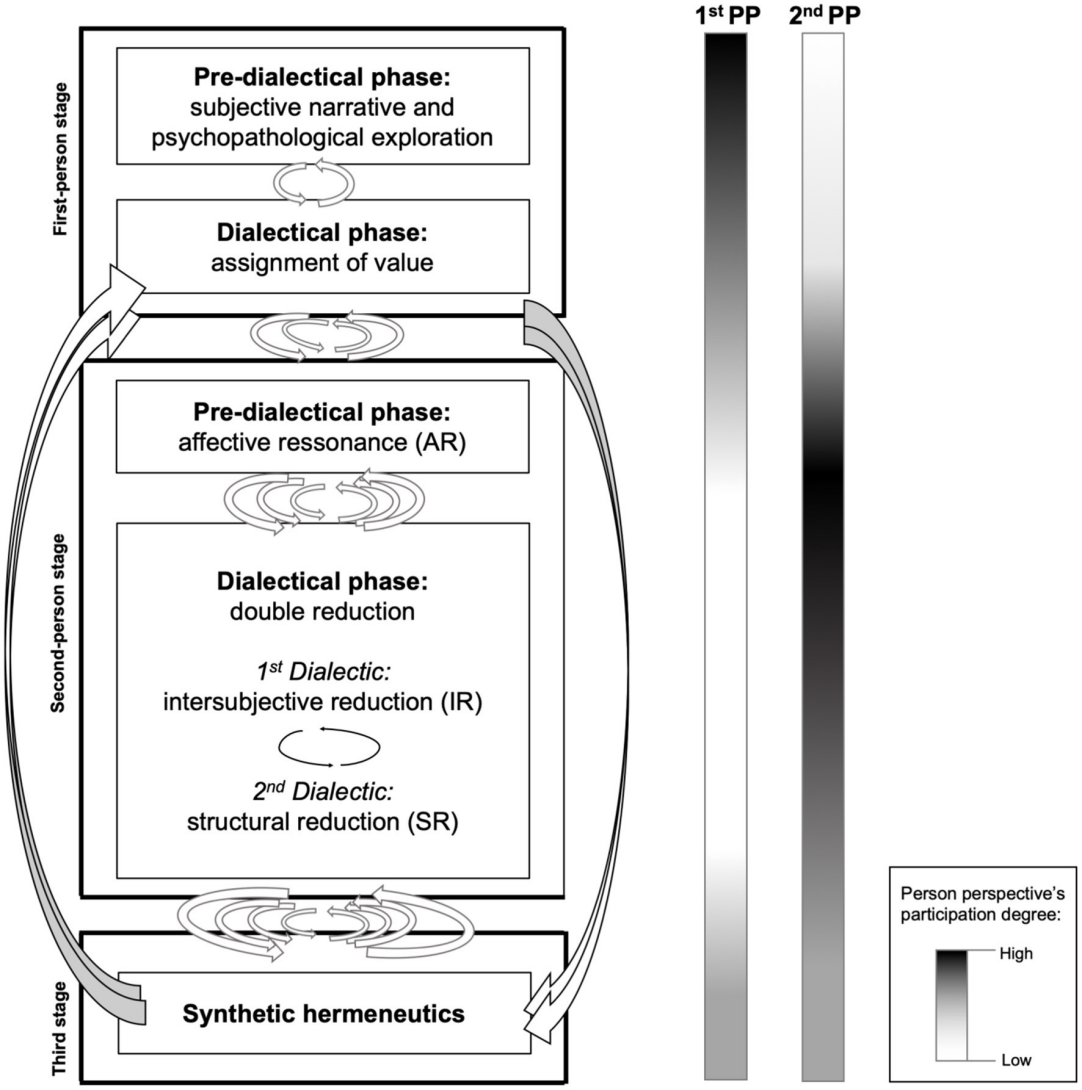
The stages of dialectical phenomenological diagnosis, with increasing degree of complexity, from top to bottom. The columns on the right indicate the degree of participation of each person at each stage.

We propose a three-stage diagnostic methodology, in which each stage is internally connected by specific dialectics. The first two stages are divided into a pre-dialectical and a dialectical phase. Overall, the diagnostic process progresses in a trajectory of increasing complexity, in which knowledge obtained at one level is dialectically absorbed and intertwined into the next levels (see Figure 1). Simultaneously, from the standpoint of degree of evidence, it starts out with pre-scientific evidence drawn from a first-person perspective stage, then progresses to attain the highest degree of scientific evidence in the second-person stage. This is then followed by a stage in which the first- and second-person evidence is combined and a final DPh diagnosis is reached.

The stages follow the usual chronology of a diagnostic interview or psychiatric or psychotherapeutic consultation.

### First-Person Stage

#### Pre-dialectical Phase: Subjective Narrative and Psychopathological Exploration

The ground zero for any psychopathological diagnosis is the person's^1^ own experience of a psychological or behavioral situation they feel unable to cope with. The subjective trigger situation is an implicitly uncomfortable experience, which gradually takes on an explicit form. As they recount this experience, the patient will pick out whatever content they think is important, while the psychopathologist listens attentively^2^. When the professional comes into contact for the first time with a fellow human being who has sought clinical help because of some experience they cannot tolerate or process, the initial step is to begin a **semi-structured** (13) or **open** (14, 15) **psychological interview**^3^. The excessive predetermination of any topic should be avoided, and the flow, rhythm, spontaneity and emphasis of the discourse should be respected (17). Psychopathologist and patient begin a conversation—usually led by the former, but not necessarily—which is gradually allowed to progress along paths that cannot be foreseen, although it is usually guided by a degree of thematic investigation into the experiences that led the patient to ask for help. Whenever necessary, the psychopathologist must intervene, requesting details of a particular experience or enquiring about some point that the patient did not value in their spontaneous speech but which the psychopathologist deems to be of diagnostic value based on their intuition of certain typical psychopathological characteristics. Particularly in the semi-structured interview, the psychopathologist must blend questions into the patient's narrative that help in the construction of the diagnosis. However, these questions should never stem the natural flow of the interaction, which should be commanded by the patient.

Narrativity is a fundamental for initiating access to another's subjective experience (13, 18, 19). The way a patient articulates their narrative should merit the psychopathologist's attention just as much as its content. Even though a patient can be expected to give a more or less concatenated narrative of the facts that brought them to the consultation (or, in the case of follow-up, the themes to be dealt with on that specific occasion), this is not always the case, since both verbal and written accounts depend on each patient's expressiveness and capacity to articulate themselves. Situations where the discourse lacks directionality are loaded with meaning for the psychopathologist (20). In non-psychotic conditions, weak directionality in spontaneous speech may indicate, for example, difficulty on the part of the patient in facing their own issues. Or alternatively, their narrative may bring up highly dramatic events which, however, no longer have any affective relevance for them. Such is the case, for example, of histrionic individuals, who will often begin the first interview by telling the psychopathologist about violence they have suffered throughout their lives. However, genuine and deserving of respect and attention this violence may indeed be, it may only serve the function of coloring the patient-psychopathologist relationship, lending a strong tone to the diagnostic setting. What actually motivated the patient to seek help may have nothing to do with these past facts. Meanwhile, in more severe conditions, the spontaneous subjective accounts of schizophrenic people are marked by a total lack of structure into logical discourse, with this very lack of structure offering, right from the start, an important clue for the professional in their diagnostic endeavor. There are yet other situations in which the ineffability of an experience can itself hamper any attempt to express it in the form of a clear narrative.

The expert psychopathologist knows that while they are paying attention to *what* the patient says, they must also observe the bodily signs that accompany the narrative: eye movement, tone of voice, flow of speech, position in the chair, even their attire and walk. Any dissonance between their account and these non-verbal indicators can itself be taken as a diagnostic clue. For example, a report of extreme anxiety accompanied by fear of losing control of one's own mind, or fear that the nature of the world is changing may be part of quite different experiences. When speaking of such things, an anxious phobic patient will display visible tension on their face and touch on the subject in a haphazard manner, as if the mere act of mentioning it could trigger some dreadful event. They may perspire, their hands may be restless, and their facial expression may convey a craving for protection. However, the same subject referred to using the very same words borne of a different experience, say, involving psychosis, will be delivered in a manner that is more distanced, more inward-looking, less directed toward the psychopathologist. In this case, they will try to get the psychopathologist to give them some explanation for what they are going through rather than appealing to them for protection.

#### Dialectical Phase: Assignment of Value to the Experience

The subjectivity of a narrative does not make it devoid of scientific validity. This is the case not only because first-person experience is ultimately what justifies the entire field of mental health, but also because it is in the narrative that the patient assigns value to their altered experience. For example, in describing their experience of sadness or despondency, an individual may evaluate it either as the result of brain disease or as moral weakness on their part. It is to be assumed that these different value assignments are underlaid by different attitudes toward psychiatric treatment. The assignment of value often appears implicitly in a subject's narrative or it is responsible for the way they frame their experiences. For this reason, it is not always easy to ascertain it without asking the patient directly. This is why the skills and other elements of values-based practice are essential to the phenomenological diagnosis of the kind we advocate in this paper (21, 22). The clear and direct investigation of the values a person attributes to their disorder is part of contemporary best practice in mental health (23, 24).

We should not understand the subjectivity of the narrative as being endowed with absolute objectivity for the narrator themself, as if it were, for example, a report of a foot fracture. Every narrative under a mental state is already a sort of exercise in hermeneutics, and so also contains a dialectical relationship within itself, centered on dialogue between the lived experience and the value that the narrator gives to it. For this reason, the first dialectic in psychopathological diagnosis is the dialectic between an altered experience and the value assigned to it by the person. The outcome of this first-level process is a narrative of an experience of suffering, which the person values as being fit to be addressed by the mental health corps of their society. The validity of this step lies in the subject's capacity for self-reflection and linguistic expression. It also requires the subject to have some cultural assimilation of current scientific categories in order to share their experience in a minimally comprehensible manner. Besides that, this first level also enables the psychopathologist to gain a preliminary overview of some experiences which may be diagnosed according to their corpus of categories and practices. Despite the obvious relevance of this step for diagnosis, it still falls short of a genuine DPh diagnosis.

### Second-Person Stage

While invaluable, first-person material cannot serve as an independent method of psychopathological investigation (25). It would be sufficient if subjectivity were not grounded in pre-reflexive intersubjectivity. It would also suffice if there were a clear and exclusive definition of a mental disorder from the person's own account. However, this is by no means universal. In psychiatry's most canonical disorder, schizophrenia, what a patient may report as a real fact is often understood in their societal context as an alteration or a delusion. In such cases, the attestation of others is necessary as validation of any pathological experience. Therefore, the dialectical inclusion of another—in this case, the psychopathologist—is a fundamental step in the diagnostic process^4^. Access to the patient's subjectivity is primarily an empathic effort on the psychopathologist's part to build up a representation, transposition and analogy^5^ of their experiences. However, putting oneself in the patient's shoes, despite its importance, is not enough of itself to take account of the complexity of the way the patient exists in the world (29), because any knowledge obtained from doing so is still born from a non-dialectical approach between the psychopathologist and the patient. To achieve this dialectical step, a methodology must be employed that fosters a deep intertwining of both patient and psychopathologists (30–32). To this end, we propose a phenomenological procedure designed to enable a clearer observation of the patient-psychopathologist dialectics.

#### Pre-dialectical Phase: The Examination of Affective Resonance

The examination of affective resonance (AR) is the first intersubjective evidence from which a DPh diagnosis is made AR, under the various names it has received throughout the trajectory of psychopathological thought, has always been prized as a fundamental precondition for a diagnosis (10, 33–41). By AR we mean a personal experience of the psychopathologist that stems directly from the interpersonal foundations of mental reality^6^. Indeed, it is not by chance that this interpersonal phenomenon draws its metaphorical origin from music. In music, the determining factor of the listener's reception and appreciation of the beauty of musical art is the sonorous phenomenon of resonance. The sonority of music, by touching and directly involving the listener, produces a kind of spiritual unification with the piece. In music, the listener and the piece of music intertwine, harmonizing in correlated emotional states. Similarly, interpersonal interplay involuntarily brings forth a number of feelings, thoughts, emotions and reactions.

The AR experience can manifest itself in two distinct ways. In consonant AR, there is a unification of the patient's reported feelings with the psychopathologist, while in dissonant AR the feelings evoked in the psychopathologist are distinct or even opposite to the ones reported by the patient. Let us look at an example of consonant AR. If we get deeply involved in an interview with a depressive patient over the course of minutes or hours, we are drawn to partially match the weight and sobriety of their pre-reflective experience of the world. Their entire depressive being “vibrates in unison” (34) with us, summoning a depressive feeling that takes us over and partially matches their reported suffering.

AR is therefore a primordial phenomenon of the intersubjective constitution of the world (10) and reveals itself in the investigator while in direct contact with of some of the patient's experiences. It is, so to speak, a rough version in the psychopathologist's consciousness of the way the experiences are organized in the patient's consciousness. For this reason, AR is the first element upon which the dialectical steps of a DPh diagnosis are built. These experiences felt by the psychopathologist are the most directly valid psychopathological object they access and therefore constitute raw data of indispensable diagnostic value (13, 42). However, since it is but one element of an intersubjective relationship, it needs further methodological polishing if it is to offer the validity required for a fully-fledged phenomenological diagnosis. The first step in this refinement, presented below, involves adding a dialectical dimension to the elementary AR stage.

#### Dialectical Phase: The Double Reduction

##### First Dialectic: Intersubjective Reduction

Reduction is a technical term which is widely used in phenomenological philosophy, especially by its founder, Husserl. In general, it indicates a methodological procedure aimed at achieving deeper access to reality. Through reduction, the objects of reality are, as it were, purified so they may be identified in their fundamental, essential characteristics, and no longer in the secondary forms through which they appear to everyday, non-scientific consciousness. It is through reduction that phenomenology acquires its particular philosophical and scientific characteristics.

Since reduction is designed to reach the primordial nature of reality, it must entail a definition of what this primordial nature of reality is. In philosophy as well as in psychology and phenomenological psychopathology, this primordial and fundamental region is intersubjectivity, which can be investigated in its pre-reflexive (sometimes called transcendental) dimension^7^. The crucial moment in the history of phenomenological thought in which the intersubjective nature of each individual consciousness is clarified occurs in Husserl's Fifth Cartesian Meditations (44). There, the author argues that every individual consciousness is never isolated, and that its very existence depends on an insertion in a connected web of other individual consciousnesses, so that the idea that, for instance, a thought of mine is only mine becomes fictitious. A common example of this is language: when we speak, although the ideas narrated are ours, the whole form of our language is determined by rules of semantics and syntax dictated by the linguistic community to which we belong.

For this reason, it is the ultimate goal of the psychopathologist. The methodology for accessing this core dimension is called **intersubjective reduction (IR)**. IR is the first reductive step of two required to make a DPh diagnosis.

Reduction is one of those procedures that are easier to perform than to define (45). Below, we summarize its *modus operandi* and give some examples to convey its practical significance^8^. The psychopathologist must employ a particular existential approach in order to successfully carry out IR, as follows. They must experimentally detach themself from their own experiences to bring forth what they have experienced implicitly thus far in their relationship with the patient (12). IR enables an upward dialectical synthesis from the first-person contents narrated by the patient to the original dialectical ontological level in which the world is constituted intersubjectively (10). With this procedure, the subject of knowledge paradoxically isolates him/herself from the intersubjective world to better understand it [(46), p. 38–39]. IR is properly the most radical dialectical movement throughout the whole DPh diagnostic process, since it is an existential movement which thoroughly challenges the ordinary experience of the psychopathologist. For example, the psychopathologist needs to annul their own discomfort in relation to the patient being examined or to detach themself from their own feeling of affection for her or him, or to attenuate their will to cure a given patient, so that they may ultimately, on a higher dialectical level, do it again more effectively. This efficacy is brought about by the fact that, with this annulment of the self, this self-imposed form of soft derealisation, their consciousness can methodologically contemplate the essential basic forms that constitute the anthropological disproportion they aim to identify. It is an active rupture of the threads that bind the human to the real so that this real can appear more clearly. This transformative action by the psychopathologist on their own self goes far beyond any philosophical, intellectual action (from which it originated), since it acts directly on the very threads that link them to others, like love, revolt, pity, solidarity, etc. In short, the psychopathologist seeks to abandon all their links with reality to seek reality itself at another level. The final dynamic of this complex process of reduction similarly exhibits a dialectic form through opposing and alternating movements of detachment and new approximations with otherness (47).

To perform this step, the psychopathologist must simultaneously pay attention to both their own subjective contents and their patient's. This makes reading oneself, in a way, equivalent to reading the essence of something that occurs in another, a phenomenon that Calvi calls “mimetic praxis” (48). Classic and contemporary authors in phenomenology have pointed this out. In the classical era, Binswanger reminded us that “the (investigator) observes himself during the act of perceiving” [(49), p. 300]. More recently, Ballerini highlighted that “whatever our therapeutic approach, we must let ourselves be guided by attention to and study of the patient's internal experience and our resonance with it: that is, observing the other's subjectivity while observing one's own” [(50), p. 35] (emphasis added).

IR examines the dynamic characteristics of this simultaneous assessment, aiming to obtain a vision of the essence of the anthropological disproportion that is at the pre-reflexive foundation of the patient's altered experience. It is from the intuition awakened by the simultaneity of two subjectivities in contact that the essential anthropological disproportion becomes apparent. An example will better clarify this point. To return to the typically histrionic patient outlined above: in the course of their subjective account, the effort we make to know their experiences in detail gradually and imperceptibly transforms us, as psychopathologists, into a powerful person. This is not sheer seduction. More often than not, it is just a diffuse mode of conduct (51–53), in which the psychopathologist does not even know how they arrived at the subjective state they reached. In this final state, they feel strong, self-assured, endowed with the power to solve the patient's problem, not infrequently taking on a paternal role before the patient. This simultaneous finding of one subject reporting weakness while the other imperceptibly turns into the strong and ruling pole of the situation is typical of histrionic anthropological disproportion (54). This typical histrionic anthropological disproportion must be distinguished, for example, from what occurs in the presence of a schizophrenic person. Let's do a thought experiment in which the patient's narrative was identical to that of the histrionic patient's, but is delivered by a patient in the early stages of a psychotic condition, who reports their unpleasant experiences in terms of extreme anxiety and fear. In the course of the interview, as the psychopathologist allows themself to enter into AR with the patient, they find their mental state differs from the one they experienced with the histrionic patient. They feel overcome by an enquiring attitude about the subjective state experienced by the patient, have difficulty in fitting it into their own experiences, find there is a mismatch between what they are used to living in their daily relationships and what the resonance with the patient provokes. They attempt to imagine what the patient's altered experiences may feel like since they cannot find them in their own inner self; in short, they experience the discomfort of being in the role of an investigator of unexplored frontiers of human life, which are usually given the name of psychosis.

A psychotic experience can only be assessed scientifically for the purpose of subsequent validation as a scientific category if it resonates affectively in the psychopathologist^9^ and is consolidated into a specific anthropological disproportion (or structural alteration, as we will see below), whose main manifestation is strangeness. This is what is given the semiological name of delusion. Kimura attributed schizophrenic disorders to an alteration of the *aida*, a Japanese word for describing the sense of “inter” that occurs in human relations (55). This *inter* is the existential site, the intersubjective structural *situs* in which the matrix of intersubjectivity occurs. It is worth reiterating that when two people enter into resonance, they do not do so with all parts of their person, but only with some of them, those that depend on the conditions of the encounter. It is only in severe psychopathological conditions that the mental alteration colors all intersubjectivity; in mild conditions, in which large sectors of existence are preserved, intersubjectivity may not reveal the core of the complaint, making more than one perspective on the same pathological experience possible and more than one source of knowledge necessary to ensure the validity of the first-person account as a psychiatric diagnosis (We will come back to this at the end of this article, because there are some consequences for the psychiatric diagnosis of this condition). For example, we can assist a depressed patient without the AR and subsequent IR being dominated by depressiveness. In such cases, the first-person perspective gains status as a primary element for the diagnosis, even if this, in our view, makes for a less valid dialectical diagnosis, as we will see below.

IR qualifies the psychopathologist to identify the essential intersubjective core of psychopathological categories. All mental illnesses, whether psychotic or not, can be addressed and understood from the notion of anthropological disproportion (10). This diagnostic recognition is achieved, methodologically, by the exercise of comparative imaginative variation. The psychopathologist, while directly intuiting the essence of the intersubjective disproportion (or structural alteration) that emerges in their experience with the patient, tries to perform a comparison between the disproportion they have just intuited and the other disproportion that they know from clinical experience. This highlights the importance of specific training for performing DPh diagnosis, since it is ultimately rooted in a recognition of modifications which builds on clinical experience. A DPh diagnosis of pre-reflexive intersubjectivity is thus a diagnosis closely tied to the *a priori* categories of phenomenological psychopathology. It depends less on total objectivity of the objects of the world and more on an intersubjective objectivity, sedimented in a scientific tradition and organized in scientific categories shared and understood by a community of peers (56).

Anthropological disproportion emerges as a scientific result of the observer's activity in producing their methodological reduction. This activity, however, has peculiar characteristics. The psychopathologist must remain in a contemplative attitude (35) in order to “summon” the manifestation of disproportion by allowing the intersubjectivity of the psychiatric interview to flow. This actively contemplative attitude must be maintained until the essential intuition of an anthropological disproportion gradually emerges in their consciousness in a clear way. Not infrequently, DPh diagnosis at this level is revealed even without an exhaustive inventory of the patient's subjective states or an objective evaluation of their mental or cognitive functions. The psychopathologist is thus only partially active in the interventions they make during the patient's narrative, guiding them along some paths that, based on prior experience, they deem to be more fruitful for the emergence of the anthropological disproportion, as already highlighted in stage 1. Ultimately, the essential intuition of an anthropological disproportion emerges as the fruit of a spontaneous reductive movement, whose primary source is independent of the wills of patient and psychopathologist, giving it a less voluntary and more structured nature than the first-person narrative. There is something that arises in their contact, a third term (57) that defies the parties' control and which organizes the structure of this intersubjectivity. While the patient is presenting us with the contents of their complaints and we are listening to them, both of which are voluntary in nature, a movement of intersubjective attuning takes place. This attuning is, ontologically speaking, more important in terms of scientific validity than the voluntary acts themselves and is therefore more valid as well (58, 59). The ultimate source of this spontaneous movement which enables the experiences of the patient and psychopathologist to evolve is also dialectical. As Jaspers states in his General Psychopathology, dialectics is the form of movement [(27), p. 340]. Thus, regardless of the willpower of the parties involved, the dialectic of intersubjectivity itself continues to produce the movement that will ultimately be taken as the object of analysis by the psychopathologist.

The importance of the essential intuition of anthropological disproportions for diagnosis is most evident in situations where there is a discrepancy between the patient's narrative and the AR. Let us imagine a case, new to the psychopathologist's experience, in which the patient's family complains that he/she is lying about certain symptoms. In this situation, the psychopathologist is faced with a dilemma: either they must believe the patient and take on the risk of potentially being misled by lies that divert them from the therapeutic goals, or they must discredit the patient, severely compromising the therapeutic bond. In this case, for the purposes of diagnostic validity, the psychopathologist cannot rely fully on the subjective accounts they are being given. And it is at this point that the paramount importance of making an intuitive diagnosis of the anthropological disproportion becomes apparent. Only the results of the IR will confirm or not the preliminary diagnostic conjectures of the case. If we develop our hypothetical case, let us imagine that throughout their account the patient narrated facts that seemed somewhat fanciful and unreal and told them in such a way as to shock the psychopathologist. If there is any untruth in their account, it will emerge in the intersubjective dialectic. In such a situation, the search for the psychopathologist's attention is what seems to drive the patient's speech. Examining the AR, the psychopathologist would report a feeling that the patient exaggerated or dramatized their suffering. In this case, we are closer to an intersubjective disproportion of a histrionic nature, as mentioned earlier (which can only be confirmed, of course, after more precise and longitudinal knowledge of the patient). On the other hand, if mistruth emerges essentially from irresponsibility with words and the depth of communication, the characteristic of the anthropological disproportion will be different. Let us imagine a picture of euphoric hypomania. Subjectively, the patient feels comfortable with their account, indifferent to what the psychopathologist might think of them. They are cheerful, agitated, filled entirely by their own subject matter. The psychopathologist, in turn, may notice that the rapport in the relationship is contagious, even if the patient's interaction is clearly inappropriate. This concurrence of indifference to the truth of the accounts combined with a pleasurable experience for both sides is typical of some euphoric hypomanias. There is a significant amount of evidence that this strategy can also be validly used for the diagnosis of several distinct disorders (60).

At this level, the diagnosed object is the intersubjective anthropological disproportion—the most valid raw source material for a DPh diagnosis, though not yet formulated in a minimally scientific form, which can only be achieved through a second reduction. Let us now explore this second validation step.

##### Second Dialectic: Structural Reduction

So far, we have emphasized the pre-reflexive characteristics of intersubjectivity, and have raised it to the status of the first pillar of validity for a psychiatric diagnosis. However, although intersubjectivity is at the basis of the constitution of reality, it is not, of course, its totality. Consciousness is defined by the dialectical articulation of other pre-reflective dimensions, such as temporality (or pre-reflective lived time), spatiality (or pre-reflective lived space), identity, embodiment, etc. (59). The result of all these dialectics is the pre-reflexive structure of consciousness. The structure of consciousness can be accessed through a new methodological reduction, called **structural reduction** (SR), in which the other pre-reflexive dimensions are dynamically introduced to the phenomenological analysis. This procedure is composed of the simultaneous identification of the distinct dialectics, both in their reciprocal relations with each other and within their component parts (61). The movement that targets this dialectical totality begins with what has already been obtained up to this point in time, reappraising the material already acquired and supplementing it in order to produce a higher-level synthesis.

This new level of access to the other builds on the methodology used in the previous step. It is, however, a new reduction, since it is carried out by a distinct cognitive act in relation to the patient's consciousness. The SR extends the methodological procedure performed in the IR to dimensions that are not directly graspable, but can be accessed by a new kind of cognitive act (62). An example will help clarify this difference. We capture directly, by an immediate intuition arising from intersubjective contact, that someone is sad. However, we do not capture directly the dimension that this sadness takes in the totality of the patient's consciousness, that is, its spatiality, or even how much this sadness floods the patient's present time, making them blind to future time. However, we can deductively reconstruct the pre-reflective temporal and spatial value of these experiences.

To further illustrate the dynamics of this synthetic step, let's take the example of an aggressive patient. When confronted with a patient who takes aggressive attitudes in the interview, the psychopathologist would detach themself from their own experience of anger and would try to identify (i) how the anger is situated in the interpersonal relationship (IR) and (ii) how the essence of anger manifests itself in the totality of the patient's consciousness (SR). The decisive element of the SR is the examination of how the experience of anger is dialectically based on intersubjectivity and the other pre-reflective dimensions of the patient's consciousness (63). For example, in the case of aggression arising out of psychotic strangeness, the IR can identify how the anger experienced by the psychopathologist does not completely take them over, leaving room for an experience of some strangeness. It is as if they did not truly “believe” their own anger, and thus wondered whether the patient's own experience may also arise from some strangeness or emptiness in their field of consciousness.

With this first intuition in mind, the psychopathologist performs the structural reductive step. In it, they can, for example, reduce the patient's anger to its pre-reflective spatial dimension, understanding it as an expression of psychotic fragmentation that, experienced as a loss of the normal profiles of reality, produces a kind of reactive aggressiveness. In the same way, they can reduce aggressiveness to its identity dimension, understanding it as stemming from the patient's inability to experience themself as an independent person and therefore forever warding off the threat of invasion and existential extinction.

The SR is the most complex and comprehensive portion of the phenomenological diagnosis and the one that depends most heavily on the psychopathologist's expertise and ability to handle phenomenological concepts. Since it is based on an act of structural deduction of the whole of the patient's consciousness, it is more distant from direct contact with the patient than the knowledge directly obtained by the IR. Therefore, for the proposed diagnosis that stems from it to be validated, its capacity to translate the patient's experience must be confirmed dialectically by them. Though this step is more vulnerable to analytical imprecision, it is crucial to transcend the immediacy of direct intersubjective knowledge and thereby enable the acquisition of a higher-level phenomenological diagnosis.

SR requires the psychopathologist to draw on the entirety of their theoretical and practical baggage along with their knowledge of the biographical, family, social and cultural context of their patient in order to clearly discriminate the pre-reflective structure of the patient's experience. Structural diagnosis is, so to speak, a sublational synthesis between the content narrated by the patient and the forms intuited (IR) or deduced (SR) by the psychopathologist. It is therefore a diagnostic level that takes time, because it requires in-depth, coherent knowledge of the individual and their altered experience. The greater this knowledge, the smaller the purely interpretative dimension of the patient's life. It is a methodological procedure based on a pre-linguistic intuitive and deductive act that is communicated primarily by non-linguistic means. We believe that in this dimension, narrativity plays a secondary, albeit indispensable, role. The level at which the SR takes place, combining intuition and deduction, is a silent region of reality, the “sphere that precedes language and thought” [(64), p. 15] in which existence is rooted and from which language retrieves only the most communicable part. Every linguistic interpretation is a plain-language hermeneutic reconstruction that is even further removed from the primary evidence of knowledge. Therefore, in the necessary dialectical passage from this level to the level of the plain language of ordinary and scientific discourse there is a degree of loss of validity, even though this is essential for establishing the social value of a science, as we shall see below.

It is at the structural level that the most elaborate and complete form of scientifically pure DPh diagnosis is given, although not yet in its definitive form. For this reason, we should now pause to briefly go through what meanings diagnoses at this level may have. The second dialectic level enables us to divide mental changes into two groups, according to the layer of existence in which the change occurs, determining two synchronous but distinct objects of knowledge. There is a hierarchical relationship between the two types; that is, the first level can occur without the second, as it is more superficial, but the second must contain the first, as it is more fundamental.

- Anthropological disorders. The examination of the dialectical relations between the anthropological disproportions determines the anthropological level of the phenomenological diagnosis (65). At this level, the alterations are of a quantitative order, so there is no alteration of the shared constitution of reality. The psychopathological object of this level are pre-reflexive anthropological disproportions of existence—temporality, spatiality, intersubjectivity, corporeality, identity, etc.—in their reciprocal dialectical relations (66). It includes all non-psychotic disorders, from so-called neurotic disorders to personality or behavioral disorders such as substance misuse (67). Anthropological disorders arise when one of the pre-reflective constituents of experience gains hegemony, configuring the anthropological disproportions typical of each disorder. For example, in melancholia, existence is impaired by an excessive proportion of prescribed roles vis-a-vis the roles creatively exercised by the self (68). In contrast, in compulsions excessive value is placed on the partial functions of an object in relation to the healthy participation in the meaningful whole of existence; e.g., food ceases to be part of nourishing oneself to become an end in itself, in a disproportionate sense.

- Structural disorders depict psychiatric conditions in which there is impairment of the intersubjective constitution of reality. The paradigm of mental disorder in which there is a loss of the shared constitution of reality is schizophrenia. Structural disorders refer to the regions of consciousness where the intersubjective constitution of reality is not possible. Given that the co-constitution of reality is a prerequisite for the dialectical interplay of pre-reflective dimensions, a structural diagnosis homes in on regions where dialectics are absent. The non-existence of dialectics manifests itself in consciousness as rigidity, most typically in the form of delusion (69). However, a structural diagnosis is somewhat more complex, since it must take into account the findings from the anthropological plane. As such, a synthetic structural diagnosis of schizophrenia must retrieve the dialectics that exist between existential zones in which there is a loss of intersubjectivity (psychotic zone) and zones in which experiences remain healthy (zone of dialectally organized anthropological proportions). After all, a person with schizophrenia continues to experience all the hardships of normal life, although these are heightened by the psychotic fracture. This synthetic structural diagnosis captures both the essence of the qualitative alteration and the quantitative meanings it denotes. This alteration is also what indicates the presence of pathological experiences in the form of phenomenological compensation (70).

Briefly, we can say that a phenomenological diagnosis of the second dialectic (arising out the sublational synthesis between the intersubjective and structural reductions) is a **positional diagnosis** (10). It indicates what the “pure” pre-reflexive structural position of the consciousness is in reality, indicating its alterations and dialectical dynamism (or lack thereof). In order to complete the cycle of phenomenological diagnosis, a new and final synthesis must be carried out, in which the diagnosis takes on a new meaning, as we will see below.

### Third Stage: Synthetic Hermeneutics

Hermeneutics is the last stage of the phenomenological diagnosis, in which all the previous levels are brought together. Though it is the final sublational synthesis of the previous dialectical moments, it should not be considered the most valid and precise part of the diagnostic process, but simply the most open and shared stage of the DPh diagnostic process. It is less scientific in terms of its in validity, but closer to the real life of individuals, marked by ambiguity and indeterminacy. Hermeneutics corresponds to the moment when interpretations are included in the diagnoses, turning them into categories of ordinary life. The hermeneutic stage is understood here as the time when meaning is assigned to the mental disorder, through a shared narrative between patient and psychopathologist (71). This shared giving of meaning enables the alteration to be incorporated into the patient's biography, with its ambiguities and singularities being attributed to the effects of past decisions, temperamental tendencies, reactions to the socioeconomic context, and the most intimate and significant interpersonal relationships. Its results are usually not clearly expressed in terms of categories of disorders, but rather as biographical moments of the person who experiences mental disorders and has to live with a certain impairment of their subjective field. At the hermeneutic level, the value-based diagnosis is added to the positional diagnosis. In a process shared by the patient and the psychopathologist, it complements and enriches the understanding of the patient's values gained through the first-person processes outlined above. Ensuring that the values of the patient are never ignored or neglected is important for best practice in all areas of healthcare but especially so in mental health (72, 73).

Unlike the reductive stage, the hermeneutic stage is based on a widening of views of reality and a corresponding diminishment of the findings yielded from the reductive stages of the diagnostic method. Although this phase is, so to speak, freer and more open to intellectual creativity, it must never lose its “anchorage in reality” (74). It must be guided by the diagnostic tonality given by the reductive phase, just as a musician who improvises in a concerto must be oriented by and attuned to the overall sense of that piece of music. Also, as the knowledge obtained in the reductive phase is more valid, it limits the range of interpretation open to the psychopathologist vis-a-vis an individual's experiences.

In the way that science is currently understood, the hermeneutic stage is not a diagnosis, in the nomothetic sense of the word, but a proposed reconstruction of the facts made by the psychopathologist to organize and offer meaning to the patient's experience. It is a narrative that is shared simultaneously by patient and psychopathologist and also by the patient's naïve self-fiction and the professional's scientifically-grounded discourse. It is ultimately a way of introducing the personal narrative into a structured scientific field, and by the same token a way of introducing the perspective of a human science to personal speech.

The results of this stage are idiosyncratic, and as such the ways people value their experiences have not yet been encapsulated in scientific language. However, the few attempts to create categories for patients' interpretations of their mental disorders have proved very promising and informative, though as yet embryonic (27, 75). The development of categories to enable a scientific approach for this level of diagnosis is a fertile area for future research.

So, what do we have, in terms of content and validity, at the end of a DPh diagnosis? Broadly speaking, the DPh diagnostic process can be seen as an ascending spiral. It begins, at a preliminary and pre-scientific level of validity, with the suffering person's narrative of their own experience. This then leads to the identification of the disproportions of pre-reflective intersubjectivity, which can be seen as the stage of maximum scientific validity. Next comes a new dialectical step in which the phenomenological reduction is widened to the entire structure of the consciousness. This broadening corresponds to a small decrease in the validity obtained as primary evidence, though it does enable the purest, that is, the most scientific form of pre-reflective DPh diagnosis. This pure DPh diagnosis is what informs the identification of the basic alteration to the structure of the consciousness, but it is still too artificial for a personalized diagnosis. The level of personalization is given by a final dialectical synthesis, in which the dialogical narrative comes into play again, now endowed with a higher degree of scientific grounding. At this point, psychopathologist and patient seek to hermeneutically reconstruct all the findings obtained by the method in a coherent narrative. This synthetic positional and value-based reconstruction offers not only the diagnosis of the disorder, but also the way in which it intertwines with the patient's biography and value system, giving a new and more fine-tuned meaning to their initial narrative. This, in turn, is indissoluble from the individual's lived reality and should therefore be understood as the ultimate synthesis of the DPh diagnosis, though not the purest one in terms of the validation of its assertions.

## The Forms of Unbalanced DPh Diagnosis

In its complete and ideal form, a DPh diagnosis is a balanced mix between the clarity of the identification of the pre-reflexive forms, obtained through phenomenological reductions, and the peculiar way in which the subjective experience of the alteration is assimilated and interpreted by the patient. The scientific validity of this desirable form of diagnosis relies on the phenomenological reductions performed by the psychopathologist, and therefore, as pointed out above, requires a minimum time interval to elapse. Both the identification of the pre-reflexive changes and the creation of a shared narrative with the patient depend on the psychopathologist having advanced in their relationship with the patient. This ideal situation, however, does not always occur in everyday practice, for many reasons. Often, a DPh diagnosis has to be reached from unbalanced access to the steps of the diagnostic procedure, leading to a less-than-ideal DPh diagnosis. Since unbalanced diagnoses are inevitable in daily practice, it is crucial that the psychopathologist be able to identify their main forms in order to recognize and mitigate the risks they contain. There are three forms of unbalanced diagnosis in the DPh method. The first two are based on insufficient grounding of the diagnosis in the second person, while the third springs from exclusive second-person grounding. Let us look at them briefly.

An **oversubjective diagnosis** occurs when there is an overreliance on information arising from the first-person stage; i.e., when a diagnosis is almost exclusively based on the content provided by the patient's narrative (not yet shared with the psychopathologist). Usually, this is an unavoidable form of diagnosis for initial stages of care or for patients who do not allow the development of familiarity with the psychopathologist. In theory, the very notion of excessive subjectivity in a diagnosis could be refuted, since subjective wellbeing is the ultimate goal of psychiatry. However, because it is fully based on a subjective account, this sort of diagnosis may not be able to discriminate the core of altered experiences as understood by both the community and psychiatrists. An example of this condition is a hypomanic person who demands to be taken off their medication because it hinders their wellbeing. As a subjective diagnosis also depends on the value the patient attributes to the disorder, oversubjectivity also happens when what is demanded by the patient cannot be offered by the clinician. Imagine a hypochondriac or a fearful person who endlessly asks the clinician to cure any experience of discomfort or fear, respectively. Complete belief in this complaint as a diagnosis may lead the clinician to, for example, overprescribe medications, which would eventually become counterproductive and harmful to the patient. One must guard against an oversubjective diagnosis under conditions in which what the person experiences as desirable is not deemed so by their community. For example, unlimited belief in subjective accounts in the case of a narcissistic (and many other forms of) personality disorder could lead to this form of unbalanced diagnosis.

A **hyperhermeneutic diagnosis** is a result of overreliance on the values-based hermeneutic stage for diagnosis. This kind of diagnosis underplays the importance of identifying the structure of the disorder and directly links the patient's narrative to a free interpretation of their speech by the psychopathologist. Paradoxically, this form of unbalanced diagnosis is more likely when the psychopathologist has a richer cultural background. Hyperhermeneutics neglects both the core of a disorder and the way a patient values it. It tends to transform the diagnosis into literature alone, giving rise to a piece of rough folk psychology rather than a diagnosis, as criticized in classical times by Jaspers (27).

On the other hand, overreliance on the pure reductive phase of the DPh methodology can have the opposite effect, yielding an **overobjective diagnosis**. In this case, the clinician may feel overly confident in their diagnosis and try to force it on the patient without considering their interest in or capacity to receive the information, not to mention the value they may assigned to their experience. This diagnostic approach sees only the altered form and not the way it is experienced as a whole.

An overobjective diagnosis is more likely to happen in some mild cases, in which the altered experience may have ambiguous value for the patient. One such case could be mild melancholic experiences, which some may experience as advantageous while others may feel are disadvantageous, or else they may be experienced as advantageous or disadvantageous at different times in life. An overobjectively oriented clinician may try not only to remove the uncomfortable symptoms experienced by the patient, but also change their structure of pre-reflective experience, having a deleterious rather than a helpful effect on the patient. In this case, the cold light of pure science may serve as an instrument of dehumanization and lack of empathy, not therapeutic power.

The fact that in their everyday clinical practice DPh psychopathologist may have to operate with an unbalanced form of diagnosis in no way diminishes the importance of the method as a diagnostic tool for psychiatry. On the contrary, as we will propose below, it is precisely an awareness of these imperfect conditions that makes the DPh perspective so well-attuned to the needs of contemporary psychiatry.

## Dialectical Phenomenological Diagnosis and Mainstream Psychiatry

At this time of growing interest in phenomenology (5), before concluding this paper we would like to indicate how DPh may make important contributions to 21st century psychiatry, all of them ultimately derived from its epistemological mindframe.

A DPh diagnosis offers a valid and scientific view of the foundations of the pre-reflexive structures of consciousness, enriched by a values-based hermeneutics. The dynamics of this synthetic diagnosis show that whenever an everyday diagnosis is crafted, there are times when validity depends primarily on the patient's account (first person), the engaged observer (second person), or others more in the role as detached observers (third person). To disregard this fact is to fail to take account of the full complexity of the relationship between validity and diagnosis, with all the consequences we have witnessed and criticized in recent decades.

To illustrate this complexity, we could say that a diagnosis of schizophrenia is sometimes most powerfully validated by the person's own narrative of some strangeness, while at other times this validity stems from a second-person observed loss of shared constitution of the world (pre-reflective intersubjectivity), and at others it comes from a third-person observed incapacity of the person to develop personal projects (mostly in negative mild cases). The fact that the validity of the diagnosis is associated with a different perspective in each case does not affect the core notion that there is a central element to the validation of a diagnosis, but simply puts it in a dynamic perspective. Thus, the scientific community's long-held goal of total objectivity in psychiatry could be enriched by diverging toward a reflection on the fact that the meaning of validity varies over time.

Instead of fixed and universal validity, the experience of DPh might suggest that the mapping of this moving terrain of validity should be a primary epistemological goal of scientific psychiatry. Adding the notion of a variable validity to the foundations of the scientific psychiatric endeavor seems to be something psychiatry could benefit greatly from. Building on this, we could argue that distinct kinds of validity might serve distinct scientific aims. Just as an example, we could propose that since the outcomes of structural reduction (pre-reflective diagnosis) belong to the purest scientific form, they should be explored as the diagnostic level *per excellence* for refining phenotypes for research, as recently suggested (76). By the same token, DPh could also address the recently noted insufficiency of diagnoses for guiding clinical decision-making (4). Contemporary needs of person-centered care require a psychiatric diagnosis which goes far beyond merely assigning a category to an experience and applying a set of guidelines to it. It is crucial for this contemporary effort to offer care according to the way generally defined disorders are embodied in real people. From what we have just presented, as DPh diagnoses are based on a dialectical approach between core pre-reflective alterations and the ways they are valued by the individual experiencing them, it has all the necessary features to guide clinical decision-making, both scientific and personal.

In short, is seems reasonable to suggest that the most cogent contribution of the proposed DPh method to general psychiatry is its assertion that ambiguity and indeterminacy are a general and unavoidable feature of all diagnostic processes in all psychiatric forms. A clear and distinct third-person diagnosis is a justifiable goal of any scientific endeavor, but it should always be attuned to the essentially dynamic reality it seeks to convey. The dream of a universal third-person, examiner-independent psychiatry has not been offered by mainstream operational diagnosis systems and apparently cannot be offered by DPh psychopathology. However, this does not mean that DPh cannot contribute meaningfully to scientific psychiatry in its most important needs.

## Conclusion

In this article, we aimed to present an overview of the DPh diagnostic process. Its validity is ensured by the increasing levels of complexity it involves, bringing together several levels of dialectically accessed validity in ascending hierarchical order, with the upper level incorporating all the results obtained at the lower levels. This method starts off from the patient's own narrative, proceeds to two stages of phenomenological reduction designed to guarantee the scientific validity of the object, and concludes with a hermeneutical narrative synthesis that is dialectically composed of the patient's and psychopathologist's shared narratives. At the end of this process, the initial first-person narrative is transformed into a specific scientific object, a full DPh psychiatric diagnosis. This values-based hermeneutical level of the diagnosis is less valid than the pre-reflective structural diagnosis, but it is more appropriate as a pragmatic instrument for adjusting the diagnosis better to real patients and thus as a guide for clinical decision-making and public policymaking. We hope to have contributed to the current psychiatric agenda, doing justice to its complexity and ultimately that of human life.

## Data Availability Statement

The original contributions presented in the study are included in the article/supplementary material, further inquiries can be directed to the corresponding author/s.

## Author Contributions

GM contributed to the conception of the study, discussed, and wrote the manuscript. LF contributed discussing the manuscript. KF contributed writing the manuscript. All authors contributed to the article and approved the submitted version.

## Conflict of Interest

The authors declare that the research was conducted in the absence of any commercial or financial relationships that could be construed as a potential conflict of interest.

## Publisher's Note

All claims expressed in this article are solely those of the authors and do not necessarily represent those of their affiliated organizations, or those of the publisher, the editors and the reviewers. Any product that may be evaluated in this article, or claim that may be made by its manufacturer, is not guaranteed or endorsed by the publisher.

## Footnotes

^1^Often, the subjective accounts of people connected to the patient can also serve as sources of information for this stage. This is necessary, for example, in cases of catatonia, in which the patient is not even able to linguistically structure their complaints. For the sake of simplicity, we will refer here only to the patient's account.

^2^Explicit first-person perspective or first person metaperspective as described by Fuchs (12).

^3^The two forms of interview share one point in common, the willingness of the researcher to hear what the patient deems most important in their first-person experience. The semi-structured phenomenological form serves as a guide to the patient's prose and can therefore be seen as an initial approach to be extended by the open form of interview (16).

^4^It is very important to highlight that although we present the psychopathologist as the ultimate source of second-person evidence, this is information that can be conveyed by any lay person who is close to the patient. As such, particularly in cases of more severe mental disorders, the psychopathologist should take due, considered account of narratives offered by relevant persons.

^5^According to Jaspers, empathy is intuitive representation. This concept is used in an ambiguous way, sometimes denoting a direct procedure and other times indirect access (representation, transposition, analogy, inference, putting oneself in the other's place) to the first-person experience of the observed (26–28).

^6^AR must be distinguished from the psychoanalytic concept of countertransference, though both stem from a radical inclusion of the observer in scientific enquiry. The phenomenological system of thought seeks to establish an original, pre-reflexive coconstituted space in which both the consciousnesses of the observer and the observed are rooted. Psychoanalytic thinking, on the other hand, operates with unconscious dynamisms that give another value to the material personally experienced by the psychopathologist.

^7^Transcendental subjectivity “can be called the primal basis for all legitimacy and validity” (43).

^8^Since the general rules of any reduction are similar, the content presented below can be extended to the next reductive step unless indicated otherwise.

^9^All reductive procedures can also be founded in information given by someone close to the patient, yielding soundly valid conclusions. Here, for the sake of simplicity, we will keep the psychopathologist as the only key person in this process.
